# Preliminary stop of the TOPical Imiquimod treatment of high-grade Cervical intraepithelial neoplasia (TOPIC) trial

**DOI:** 10.1186/s12885-017-3108-9

**Published:** 2017-02-07

**Authors:** M. M. Koeneman, A. J. Kruse, L. F. S. Kooreman, A. zur Hausen, A. H. N. Hopman, S. J. S. Sep, T. Van Gorp, B. F. M. Slangen, H. J. van Beekhuizen, A. J. M. van de Sande, C. G. Gerestein, H. W. Nijman, R. F. P. M. Kruitwagen

**Affiliations:** 1 Department of Obstetrics and Gynaecology, Maastricht University Medical Center, Post box 5800, 6202 AZ Maastricht, The Netherlands; 2 GROW, School for Oncology and Developmental Biology, Maastricht University Medical Center, Maastricht, The Netherlands; 3 Department of Pathology, Maastricht University Medical Center, Maastricht, The Netherlands; 4 Department of Molecular Cell Biology, Maastricht University Medical Center, Maastricht, The Netherlands; 5 Department of Internal Medicine, Maastricht University Medical Center, Maastricht, The Netherlands; 6 Department of Obstetrics and Gynaecology, Erasmus MC Cancer Institute, Rotterdam, The Netherlands; 7 Department of Obstetrics and Gynaecology, Meander Medical Center, Amersfoort, The Netherlands; 8 Department of Obstetrics and Gynaecology, University Medical Center Groningen, Groningen, The Netherlands

**Keywords:** Cervical intraepithelial neoplasia, Imiquimod, Biological markers, Human papillomavirus, Natural history

## Abstract

The “TOPical Imiquimod treatment of high-grade Cervical intraepithelial neoplasia” (TOPIC) trial was stopped preliminary, due to lagging inclusions. This study aimed to evaluate the treatment efficacy and clinical applicability of imiquimod 5% cream in high-grade cervical intraepithelial neoplasia (CIN). The lagging inclusions were mainly due to a strong patient preference for either of the two treatment modalities. This prompted us to initiate a new study on the same subject, with a non-randomized, open-label design: the ‘TOPical Imiquimod treatment of high-grade Cervical intraepithelial neoplasia (TOPIC)-3’ study.

*Original TOPIC-trial: Medical Ethics Committee approval number METC13231; ClinicalTrials.gov Identifier: NCT02329171, 22 December 2014.*

*TOPIC-3 study: Medical Ethics Committee approval number METC162025; ClinicalTrials.gov Identifier: NCT02917746, 16 September 2016.*

## Main text

The TOPical Imiquimod treatment of high-grade Cervical intraepithelial neoplasia (TOPIC) trial started in January 2015, with the aim to evaluate the treatment efficacy and clinical applicability of imiquimod 5% cream in high-grade cervical intraepithelial neoplasia (CIN). A study protocol for this study was published in BMC Cancer in February 2016 [1]. Patients were randomized into one of two treatment arms: the imiquimod treatment arm, in which subjects were treated with imiquimod during 16 weeks, or the standard treatment arm, in which large loop excision of the transformation zone (LLETZ) was performed. An earlier version of the study included a third treatment arm: an observational arm, which consisted of ‘watchful waiting’ during 20 weeks, with the aim to evaluate spontaneous regression of high-grade CIN and identify prognostic biomarkers for spontaneous regression. The observational arm was removed from the study after 9 months to increase the inclusion rate. The current study stopped preliminary in May 2016 due to lagging inclusions.

The lagging inclusions may be explained by the very different nature of the treatment modalities and the strong preferences of women for either imiquimod or LLETZ treatment. We experienced that women have a general preference for LLETZ treatment, as it provides a fast and effective treatment of high-grade CIN. This was confirmed by a recent patient preference study performed by the author (manuscript in press). This preference study also showed that a preference for imiquimod treatment is largely restricted to women with a future pregnancy wish and that a treatment efficacy of at least 72% is desired by these women. Indeed, we experienced that only a subset of women with a future pregnancy wish wanted to participate in the study. All of these women had a strong preference for imiquimod treatment. The amount of women willing to participate was too small to achieve the intended study population of 120 in one study centre, within the time-frame of the study. For this reason, the study was preliminary stopped.

Twelve women were included in the total duration of the study: six women were included in the observational arm, three in the imiquimod arm and three in the standard treatment arm. Of the women included in the observational arm, two showed spontaneous regression to CIN1 or less. Two women showed persistent CIN2 after 10 and 20 weeks and were subsequently treated by LLETZ. The other two women quit the study immediately after randomization and were treated by LLETZ. Of the three women who were randomized for imiquimod treatment, one quit the study immediately after randomization and was treated by LLETZ. The other two women completed imiquimod treatment and both showed disease regression to CIN1 or less.

As a consequence of the very different nature of the two treatment modalities and the differences in treatment preferences among different populations of women, imiquimod will most likely not develop as a treatment alternative for all women with high-grade CIN, but may be restricted to women with recurrent lesions or a future pregnancy wish. These insights prompted us to initiate a new study comparing the treatment efficacy and clinical applicability of imiquimod 5% cream to LLETZ treatment in high-grade CIN, in selected populations of women with a preference for either of the two treatment modalities. The ‘TOPical Imiquimod treatment of high-grade Cervical intraepithelial neoplasia (TOPIC)-3 study’ is a multicenter, open-label, non-randomized, controlled study, evaluating treatment efficacy, side-effects and quality of life associated with imiquimod treatment of high-grade CIN lesions in a selected population of women who prefer imiquimod treatment instead of LLETZ. The study also aims to develop a biomarker profile to predict clinical response to imiquimod treatment. This enables selection of patients in which good treatment response is expected. The study was approved by the Medical Ethics Committee AZM/UM (approval number METC162025) and has been registered on ClinicalTrials.gov (ClinicalTrials.gov Identifier: NCT02917746, 16 September 2016). The study started recruiting patients in November 2016.

## Abbreviations

CIN: Cervical intraepithelial neoplasia
LLETZ: Large loop excision of the transformation zone
TOPIC: TOPical Imiquimod treatment of high-grade Cervical intraepithelial neoplasia
